# Brazilian consensus on the management of bleeding complications related to the use of oral anticoagulants

**DOI:** 10.1590/1806-9282.20252032

**Published:** 2026-07-31

**Authors:** Renato Delascio Lopes, Pedro Gabriel Melo de Barros e Silva, Remo Holanda de Mendonça Furtado, Ariane Vieira Scarlatelli Macedo, João Carlos de Campos Guerra, Ana Thereza Cavalcanti Rocha, Joyce Maria Annichino Bizzacch, Writing Committee for the SBTH Consensus Collaboration, Executive Committee, Renato Delascio Lopes, Pedro Gabriel Melo de Barros e Silva, Remo Holanda de Mendonça Furtado, Ariane Vieira Scarlatelli Macedo, João Carlos de Campos Guerra, Ana Thereza Cavalcanti Rocha, Joyce Maria Annichino Bizzacch, Collaborators, Alexandre Coutinho Teixeira de Freitas, Antonio Carlos da Silva Moraes, Bernardo Pinheiro de Senna Nogueira Batista, Christiane Soares Poncinelli, Fábio Guilherme Caserta Maryssael de Campos, Liana Maria Tôrres de Araujo Azi, Lucas Oliveira Junqueira e Silva, Luciana Ferreira Franco, Maramelia Miranda Alves, Mauro Goldbaum, Ricardo Caponero, Ricardo Pavanello, Rogerio da Hora Passos, Roni de Carvalho Fernandes, Sheila Martins, Suely Meireles Rezende, Tomaz Crochemore, Valquíria Pelisser Campagnucci, Venina Isabel Poço Viana Leme de Barros

**Affiliations:** 1 Duke University School of Medicine – Durham (NC), United Sates; 2 Brazilian Clinical Research Institute – São Paulo (SP), Brazil.; 3 Sociedade Brasileira de Trombose e Hemostasia, Faculdade de Ciências Médicas da Santa Casa de São Paulo – São Paulo (SP), Brazil.; 4 Einstein Hospital Israelita – São Paulo (SP), Brazil.; 5 Faculdade de Medicina da Bahia da Universidade Federal da Bahia Escola Bahiana de Medicina e Saúde Pública – Salvador (BA), Brazil.; 6 Sociedade Brasileira de Trombose e Hemostasia, Universidade Estadual de Campinas – Campinas (SP), Brazil.

**Keywords:** Anticoagulants, Hemorrhage, Thromboembolism

## Abstract

**OBJECTIVE:**

To provide practical, evidence-based guidance for the evaluation and management of bleeding complications associated with oral anticoagulations, including vitamin K antagonists and direct oral anticoagulants, with emphasis on reversal strategies, supportive care, and safe resumption of anticoagulation.

**METHODS:**

This Brazilian multidisciplinary consensus was developed by the Brazilian Society of Thrombosis and Hemostasis (SBTH) based on contemporary international guidelines and the best available scientific evidence. Recommendations were reviewed by a multidisciplinary panel of experts and adapted to the Brazilian clinical setting and local resource availability.

**RESULTS:**

The document proposes a structured stepwise approach for the management of oral anticoagulation-related bleeding, including initial stabilization, bleeding severity assessment, laboratory evaluation, temporary interruption of anticoagulation, and indication of reversal or hemostatic therapies in patients with major bleeding and significant circulating anticoagulant levels. Specific management strategies are detailed according to anticoagulant class, including idarucizumab for dabigatran and andexanet alfa or prothrombin complex concentrates for factor Xa inhibitors. The consensus also emphasizes multidisciplinary follow-up and individualized decision-making regarding anticoagulation resumption after bleeding control.

**CONCLUSION:**

Management of bleeding in patients receiving oral anticoagulants requires rapid clinical assessment, severity stratification, targeted reversal strategies, and multidisciplinary care. Individualized reassessment of thrombotic versus hemorrhagic risk is essential to optimize outcomes and guide safe reintroduction of anticoagulation therapy after bleeding resolution.

## INTRODUCTION

Thromboembolic diseases represent the main cause of mortality in Brazil and worldwide^1^. Antithrombotic drugs are those that reduce thrombus formation by inhibiting platelet aggregation (antiplatelet agents) or by acting, directly or indirectly, on the plasma phase of coagulation (anticoagulants). Among the antithrombotic therapies, oral anticoagulation (OAC) is used as a fundamental element for the prevention and treatment of several thromboembolic conditions^2-6^.

Patients with thromboembolic diseases may also have a higher risk of hemorrhagic events, which can be potentiated by the use of OAC^7,8^.

Currently, the main OAC options available in Brazil include vitamin K antagonists (VKAs) and direct oral anticoagulants (DOACs). The former inhibits vitamin K-dependent coagulation factors such as factors II, VII, IX, and X, as well as natural anticoagulants protein C and protein S.

The DOACs act on two main targets of the coagulation pathway: thrombin (e.g., dabigatran) and Xa (e.g., rivaroxaban, apixaban, and edoxaban).

The overall risk of bleeding in patients anticoagulated with DOACs is significantly lower when compared with those under VKA^9^, including an approximately 50% reduction in the risk of intracranial hemorrhage. However, serious bleeding events can still occur in patients under DOACs, although with a lower lethality rate than the bleeding events occurring in patients using VKA. For this reason, it is essential to continuously monitor and control other factors that influence the risk of bleeding, in addition to the choice of anticoagulant agent.

Several models have been developed to assess the risk of bleeding in patients using OAC according to the clinical indication (atrial fibrillation [AF], venous thromboembolism [VTE])^2-6,10,11^. However, none of them should be used in isolation to contraindicate anticoagulant therapy. These scores are useful for estimating the risk of complications and, especially, for helping to recognize and act on modifiable risk factors, aiming at preventing bleeding. Recommended models in AF and VTE include the HAS-BLED^10^ and VTE-BLEED^11^, respectively.

DOACs are generally avoided during pregnancy because of the limited safety data and concerns regarding fetal exposure^12^. Therefore, in pregnant patients with a clinical indication of oral anticoagulation, current guidelines recommend parenteral anticoagulation (preferably low-molecular-weight heparin), since it does not cross the placenta^13^. During the puerperium, oral anticoagulants may be considered in selected cases; however, in situations of postpartum hemorrhage or increased bleeding risk, temporary use of agents with shorter half-life and reversibility (such as unfractionated heparin) may be preferable. The limited evidence regarding DOAC safety in pregnancy and breastfeeding highlights an important gap in current anticoagulation strategies.

For any clinical assessment, clinicians should consider the balance between thromboembolic events and the risk of bleeding. Because of the overlap between the factors associated with both risks, dynamic assessments at each physician visit are needed on an ongoing and individualized basis. When a bleeding episode occurs, it should be addressed promptly and according to its severity and location, irrespective of prior use of anticoagulants.

## OBJECTIVE OF THIS DOCUMENT

The objective of this document is to provide practical clinical guidance for the management of patients on OAC who progress with bleeding, based on the most recent available evidence. From the initial conduct in emergency care to specific strategies according to the location of the bleeding, including the decision on the use of specific reversing agents (such as idarucizumab or andexanet alfa) and/or non-specific hemostatic therapies, will be addressed. In addition, the document discusses the clinical criteria for the safe reintroduction of anticoagulation after control of the bleeding event.

## METHODOLOGY OF THIS DOCUMENT

This document was developed by seeking the best available scientific evidence^14,15^ and aims to guide clinical practice, but does not exempt healthcare professionals from their care responsibilities in the individual care of their patients. The recommendations given by this document should also be adapted to the local reality and availability of procedures and medications in each institution, and preferences of the patient.

The Brazilian Society of Thrombosis and Hemostasis (SBTH), through its scientific committees, aims to update this document whenever new relevant scientific evidence emerges that justifies it. The recommendations presented here were carefully prepared and submitted to the review of a multidisciplinary committee of experts, in addition to having been fully aligned with the international guidelines for the preparation of documents of this nature^16^.

Reinforcing their commitment to transparency, all authors have fully declared their potential conflicts of interest, which will be published as an annex to this document, ensuring clarity and integrity to the process of preparing this consensus^17^.

## RECOMMENDATIONS FOR INITIAL DIAGNOSTIC EVALUATION AND THERAPEUTIC MANAGEMENT OF PATIENTS WITH BLEEDING UNDER USE OF ORAL ANTICOAGULANTS

**Figure 1:**
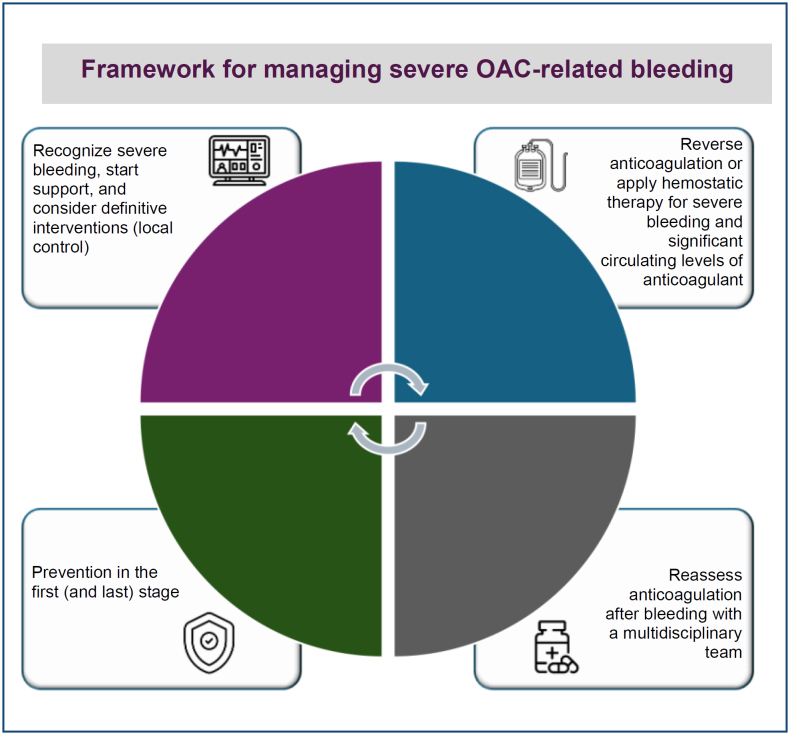
Structure for bleeding management. Adapted from Cormier and Siegal^18^.

Procedures for diagnostic evaluation and therapeutic management of patients with bleeding can be better organized and evaluated for continuous improvement of their practices in each service when multidisciplinary teams monitor bleeding patients (the so-called bleeding teams) (Figure 1).

## INITIAL CLINICAL EVALUATION

For the clinical evaluation of patients who arrive at the emergency care unit with bleeding using OAC, the initial step includes clinical assessment to define the sites of visible or probable bleeding, comorbidities, the last time of use of the OACs, and concomitant use of other medications^19^.

At the same time, the physical examination should include the evaluation of the patient's stability in terms of vital signs and perfusion.

At first, every patient with suspected bleeding should be monitored and have hemodynamic support because it is a potentially critical condition. Point-of-care ultrasound (POCUS), when accessible, is useful for localizing the bleeding site.

## CLASSIFICATION OF BLEEDING SEVERITY

**Figure 2:**
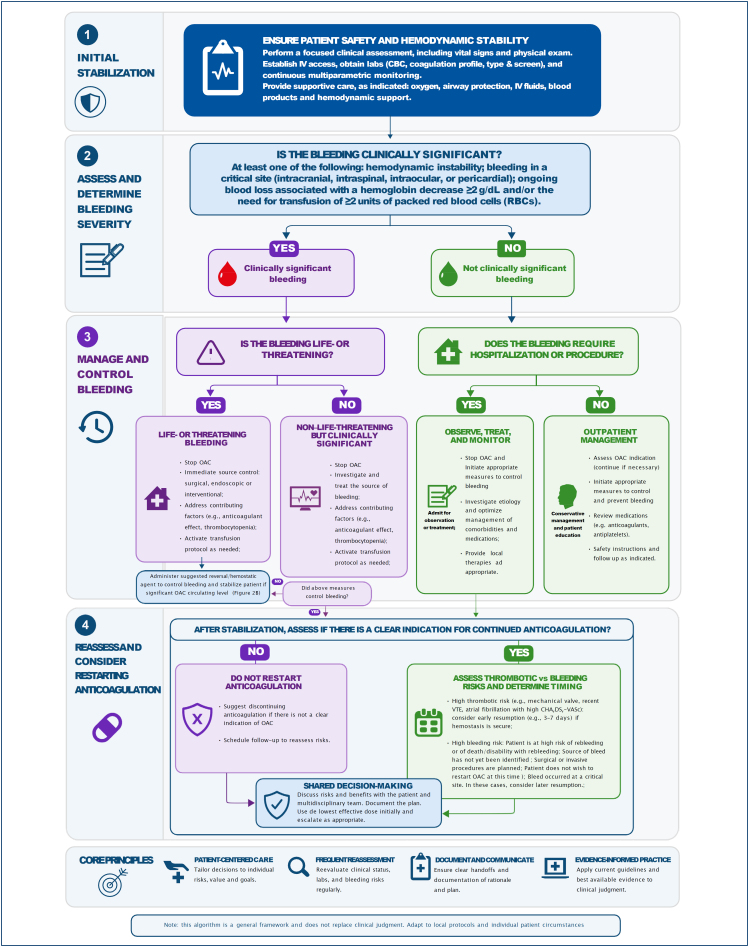
Stepwise clinical algorithm for the management of bleeding in patients on oral anticoagulant therapy^20,21^. OAC: oral anticoagulant, RBC: red blood cell, *Bleeding severity adopted in this document used variables from diverse bleeding classifications (ISTH and GUSTO) in order to make it more useful in clinical practice.

The classification of hemorrhage severity plays a central role in the management of bleeding using OAC (Figure 2).

This stratification takes into account the location and extent of the hemorrhage, the individual characteristics of the patient, and the repercussions of the event^7,8^. This evaluation is dynamic, i.e., non-major bleeding can evolve with severity criteria (e.g., hemodynamic worsening) and become major bleeding.

## LABORATORY EVALUATION

OAC can alter coagulation tests (Table 1)^22-25^.

**Table 1:** Tests altered by types of anticoagulants.

Laboratory test	Anticoagulants
PT/INR	Warfarin, rivaroxaban
(aPTT)	UFH, dabigatran
dTT	Dabigatran
Anti-X activity	LMWH, rivaroxaban, apixaban, edoxaban
Thromboelastometry	Any anticoagulant

PT: prothrombin time; INR: international standardization ratio; aPTT: activated partial thromboplastin time; dTT: dilute thrombin time; UFH: unfractionated heparin; LMWH: low-molecular-weight heparin.

In the presence of a patient with major bleeding, including hemorrhagic shock, it is essential to diagnose and treat the so-called lethal triad (acidosis, hypothermia, and coagulopathy), which has been expanded in the contemporary concept of "lethal diamond" with the addition of hypocalcemia^26,27^.

Routine tests in the initial evaluation of critical patients (e.g., arterial blood gases, lactate, complete blood count) should be performed together with tests to evaluate blood coagulation such as prothrombin time (PT) measured by INR, activated partial thromboplastin time (aPTT), thrombin time (TT), and fibrinogen (Clauss method)^27-30^.

It becomes relevant to identify hemostatic pathways that are altered, and in this sense, viscoelastic blood tests such as thromboelastometry/thromboelastography can be decisive, since they allow the differentiation between bleeding of mechanical or vascular causes, and that related to coagulopathy. The identification of hyperfibrinolysis imposes specific measures that have a major impact on the treatment success.

## BLEEDING MANAGEMENT IN PATIENTS USING ORAL ANTICOAGULANTS

### General supportive measures and care planning according to bleeding severity

The general approach was divided into sequential steps, although several actions may occur concomitantly in clinical practice (Figure 2).

1. Initial stabilization (Airway protection and ventilation optimization followed by hemodynamic resuscitation)
  Initial stabilization is essential and should also aim to prevent new complications (hypothermia, for example). It is important to note that, in the presence of active bleeding, the primary use of crystalloids is also justified because coagulation and platelet function are impaired by all hydroxyethyl starch and gelatin solutions, since they compromise fibrin polymerization and adequate clot formation^31,32^.
  Blood components may be required in cases of hemorrhagic shock^33-35^.
2. Identification of the source and severity of the bleeding
3. Temporary interruption of the OAC
  Initial interventions include the use of activated charcoal in patients whose medications have been taken within the last 4 h.
4. Specific interventions according to bleeding site (ocular, intracranial, others) and type of OAC
  In major bleeding, consider invasive interventions and specific treatments (e.g., reversal) according to the type of OAC (if drug is at a significant circulating level)^36^.
5. Care planning

For non-major hemorrhages, clinical support and close monitoring may be sufficient. In major bleeding, the decision of returning to anticoagulation should be made with a multidisciplinary team and close monitoring.

## SPECIFIC TREATMENT ACCORDING TO THE TYPE OF ORAL ANTICOAGULANT

The use of specific reversers and hemostatic agents should be done judiciously and should be considered only if two conditions are present (Figure 3A):

1. Major bleeding
2. Anticoagulant at a significant circulating level according to laboratory measurement and/or clinical data (time of last dose)

**Figure 3A:**
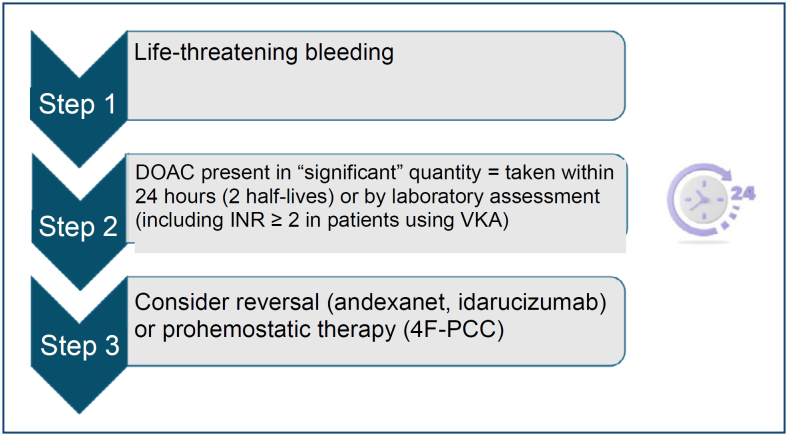
Steps before decision of using reversal/hemostatic agents. DOAC: direct-acting oral anticoagulant; VKA: vitamin k antagonists; 4F-PCC: four-factor prothrombin complex concentrate.

Consider use of reversal/hemostatic agents if a significant circulating level of DOAC is measured or suspected (<24 h) or in cases of abnormal coagulation tests (e.g., INR≥2 in patients using VKA) in patients with life-threatening bleeding (e.g., bleeding at a critical site).

Agent choices vary depending on the type of anticoagulant (Figure 3B) and should be checked in cases of major life-threatening bleeding (e.g., in a critical location).

**Figure 3B:**
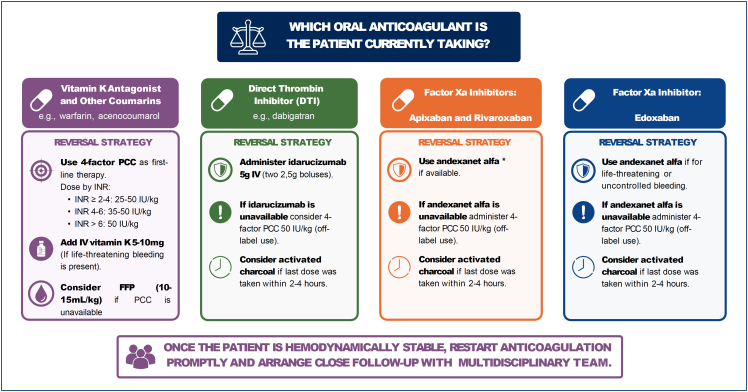
Reversal/hemostatic strategies according to the type of OAC. 4F-PCC: four-factor prothrombin complex concentrate; aPCC: activated prothrombin complex concentrate; DTI: direct thrombin inhibitor; Fxa: factor Xa; ICH: intracranial hemorrhage; INR: international normalized ratio; PCC: prothrombin complex concentrate; VKA: vitamin k antagonists; OAC: oral anticoagulant. Although andexanet alfa is available for use in Brazil, its administration should be conducted with caution, taking into account the potential associated risks. It should be noted that, in some countries, this medication has been withdrawn from the market.

Major bleeding with significant OAC circulating level^20^.

## MANAGEMENT AFTER BLEEDING CONTROL

Active and relevant bleeding is a formal contraindication to the use of oral anticoagulants^3,7,8^. In this situation, bleeding should be controlled whenever possible, and the causal factor should be corrected to avoid a new bleeding event.

Checklist before reintroducing OAC

- Confirm that bleeding has been truly controlled
- Perform effective control of the risk factor for bleeding
- Discuss risks and benefits of resuming OAC (assess the risk of delaying reintroduction of OAC and the risk of recurrent bleeding)
- Shared decision on timing for reintroduction (in cases of major bleeding)
- Multidisciplinary follow-up with an individualized monitoring plan and close monitoring of the patient (especially in cases of major or recurrent bleeding)
- Ongoing assessment of individual bleeding risk (intensify efforts to modify risk factors for bleeding)
- Continuous review of dose and type of anticoagulant used (prioritizing options with the best safety profile)

The best time to reintroduce oral anticoagulants after major bleeding is an individualized decision and fundamentally depends on the judgment between thrombotic risk vs. hemorrhagic risk^3,7,8^:

- Thrombotic risk: thrombotic risk can influence the decision about reintroducing OAC. For example, if the patient has a mechanical prosthesis in the mitral position and/or intracardiac thrombus, an earlier resumption of the OAC should be considered. On the contrary, in a patient with a history of isolated deep vein thrombosis and who was using OAC outside the acute period of the event, the resumption could be delayed.
- Risk of a new bleeding: the risk of a new bleeding also influences the decision. This includes several aspects such as: (1) site and severity of index bleeding; (2) effective control of the causal hemorrhagic factor (in bleeding due to trauma or reversible causes, there is greater safety to resume the OAC); (3) ability to manage the patient post-reintroduction by a multidisciplinary team and with close monitoring (this team facilitates an earlier and safer resumption of the OAC).

This decision about the best time to reintroduce OAC after a major bleeding should ideally be made in a shared multidisciplinary fashion.

### Follow-up by a multidisciplinary team

In addition to the medical team trained to provide care for patients who have bleeding using OAC, it is also important to educate patients taking these medications to recognize the signs and symptoms of bleeding events and to alert their healthcare provider when such events occur^3,7,8,37-40^.

Ensuring long-term safety in patients using oral anticoagulants requires shared responsibility and effective communication among all parties involved. Patients, their families, and healthcare professionals must work together to promote understanding, adherence, and early recognition of potential complications. A collaborative and well-informed approach is essential, particularly after a bleeding event, to optimize outcomes and maintain treatment continuity with the highest standards of safety.

## Data Availability

No new data were generated or analyzed in the preparation of this consensus document. The recommendations were developed based on review and interpretation of previously published scientific literature and international guidelines, all of which are cited in the reference list.
